# Bacterial catabolism of membrane phospholipids links marine biogeochemical cycles

**DOI:** 10.1126/sciadv.adf5122

**Published:** 2023-04-26

**Authors:** Linda M. Westermann, Ian D. E. A. Lidbury, Chun-Yang Li, Ning Wang, Andrew R. J. Murphy, Maria del Mar Aguilo Ferretjans, Mussa Quareshy, Muralidharan Shanmugan, Alberto Torcello-Requena, Eleonora Silvano, Yu-Zhong Zhang, Claudia A. Blindauer, Yin Chen, David J. Scanlan

**Affiliations:** ^1^ School of Life Sciences, University of Warwick, Gibbet Hill Road, Coventry, CV4 7AL, UK.; ^2^ Molecular Microbiology: Biochemistry to Disease, School of Biosciences, University of Sheffield, Sheffield, S10 2TN, UK.; ^3^ College of Marine Life Sciences and Frontiers Science Center for Deep Ocean Multispheres and Earth System, Ocean University of China, Qingdao, China.; ^4^ State Key Laboratory of Microbial Technology, Marine Biotechnology Research Center, Shandong University, Qingdao, China.; ^5^ Department of Chemistry and Photon Science Institute, The University of Manchester, Oxford Road, Manchester, M13 9PL, UK.; ^6^ Department of Chemistry, University of Warwick, Coventry, CV4 7AL, UK.

## Abstract

In marine systems, the availability of inorganic phosphate can limit primary production leading to bacterial and phytoplankton utilization of the plethora of organic forms available. Among these are phospholipids that form the lipid bilayer of all cells as well as released extracellular vesicles. However, information on phospholipid degradation is almost nonexistent despite their relevance for biogeochemical cycling. Here, we identify complete catabolic pathways for the degradation of the common phospholipid headgroups phosphocholine (PC) and phosphorylethanolamine (PE) in marine bacteria. Using *Phaeobacter* sp. MED193 as a model, we provide genetic and biochemical evidence that extracellular hydrolysis of phospholipids liberates the nitrogen-containing substrates ethanolamine and choline. Transporters for ethanolamine (EtoX) and choline (BetT) are ubiquitous and highly expressed in the global ocean throughout the water column, highlighting the importance of phospholipid and especially PE catabolism in situ. Thus, catabolic activation of the ethanolamine and choline degradation pathways, subsequent to phospholipid metabolism, specifically links, and hence unites, the phosphorus, nitrogen, and carbon cycles.

## Teaser

P-dependent phospholipid catabolism provides a valuable source of N, C and energy thereby connecting major biogeochemical cycles.

## INTRODUCTION

The microbial loop, which increases the efficiency of marine food webs through the utilization of dissolved organic matter (DOM), unifies a broad diversity of oceanic microbes including phototrophic cyanobacteria and heterotrophic bacteria and archaea (*1*–*5*). While intensively studied, it is still not fully understood how all available nutrients are shared between microbes, especially how individual nutrient cycles relate to one another. Recent studies have identified phosphorus (P) and nitrogen (N) compounds as key limiting nutrients for primary production (*6*). While several key P and N cycling enzymes, e.g., phosphatases and aminopeptidases, have been identified, evidence directly linking these cycles is limited. In the ocean, while inorganic P in the form of phosphate (P_i_) is the preferred source for microbes, organic forms are much more plentiful (*7*–*9*). Recent evidence shows the presence of a global P redox cycle with P cycling between the +5 (phosphate) and +3 (phosphonates) oxidation state, i.e., between organic and inorganic forms (*10*–*12*), which may be driven by interactions with marine carbon (C) and N cycles via compounds such as aminophosphonates (*11*, *13*).

Phospholipids are present in all life forms, generally as components of membranes which form a barrier to the external environment (*14*–*17*) but also as extracellular vesicles where they are thought to play roles in intercellular communication, nutrient acquisition, and cellular defense (*18*, *19*). In phytoplankton and marine heterotrophs, phospholipids account for around 10 to 20% of cellular P content (*20*) and between 10 and 55% of the total marine lipid pool (*21*–*23*). In seawater, cell death or cell lysis, mediated by viruses and inefficient grazing, or the release of extracellular vesicles, likely results in a massive pool of phospholipid components that can be recycled to redeem P, N, or C. Membrane phospholipids are also a valuable source of P, and many marine bacteria and phytoplankton remodel their lipids internally when P is scarce, replacing P-containing lipids with non–P-containing alternatives (*24*–*27*). The marine phospholipid pool comprises not only familiar phospholipids such as phosphatidylethanolamine and phosphatidylcholine but also many novel and unknown saturated and unsaturated phospholipids (*28*). Despite the plethora and abundance of phospholipids in seawater, there is a dearth of information on the corresponding degradation processes, especially how phospholipid components are degraded externally, transported into the cell, and catabolized by marine bacteria.

Here, using the model heterotrophic bacterium *Phaeobacter* sp. MED193 (hereafter *Phaeobacter*), a marine *Roseobacter* isolated from P_i_-deplete Mediterranean waters, we elucidate the molecular basis of how exogenous membrane phospholipid components, namely, the lipid headgroups phosphorylethanolamine (PE) and phosphocholine (PC), are acquired and catabolized. PC and PE both support *Phaeobacter* growth as the sole external source of P, with the PhoX phosphatase contributing to the extracellular cleavage of these headgroups. Utilizing proteomics, we show that growth on these P-lipid headgroups results in the synthesis of transporters and enzymes specific for ethanolamine and choline utilization, including an ethanolamine tripartite ATP-independent periplasmic (TRAP) transporter (named here EtoX), that is highly expressed in ocean metatranscriptomes. We also provide evidence that, in addition to the PhoX/PhoB-mediated degradation of PE and PC headgroups, further Pho-independent pathway(s) exist.

## RESULTS

### *Phaeobacter* can utilize exogenous phospholipid headgroups as a sole source of P

**Fig. 1.**
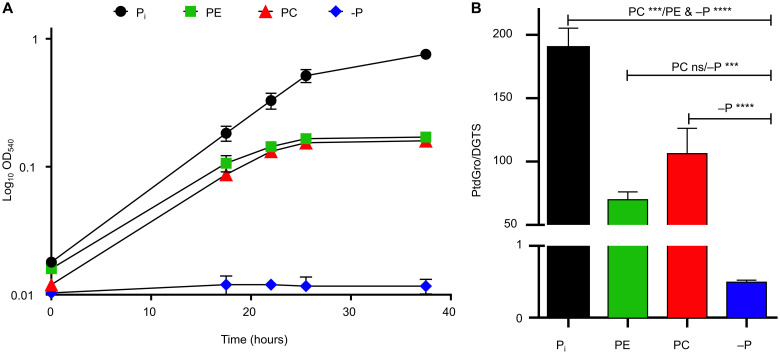
Growth and lipid composition of *Phaeobacter* sp. MED193 when utilizing phospholipid headgroups as the sole P source. (**A**) Growth (*n* = 3) of prestarved (48 hours) *Phaeobacter* sp. MED193 wild type on P_i_, PE, PC, and no P (−P) as the sole source of P. Error bars denote SD of the mean. (**B**) Ratio of PtdGro to DGTS in culture samples taken during exponential growth. Error bars represent the SD of the mean. Statistical analyses were performed with one-way analysis of variance (ANOVA) by using three replicates. *P* values: not significant (ns) > 0.01, ***P < 0.001, ****P < 0.0001; *F* value, 110.1; degrees of freedom (DF), 8.

*Phaeobacter* cells prestarved for P (for 48 hours) grew on 173.2 μM exogenous PC or PE, albeit at a lower specific growth rate than cells grown on similar quantities of P_i_ (μ = 0.255, 0.121, and 0.171 hour^−1^ for P_i_, PC, and PE grown cultures, respectively) and with a lower final cell yield (Fig. 1A). In addition, *Phaeobacter* can remodel its membrane phospholipids in response to P starvation, replacing them with the non-P betaine lipid diacylglyceryl trimethylhomoserine (DGTS) (*27*). Thus, we hypothesized that supplementing P-deplete cultures with PE and PC would attenuate these membrane lipid modifications. To test this idea, we extracted membrane lipids from *Phaeobacter* cultures grown on each P source. Forty-eight–hour P-starved cultures showed an obvious increase in the ratio of DGTS to phosphatidylglycerol (PtdGro) (Fig. 1B). Supplementing P_i_, as well as PE and PC to prestarved cell cultures slowly increased the PtdGro/DGTS ratio (Fig. 1B), confirming that *Phaeobacter* utilizes P_i_ acquired from PE and PC to restore membrane phospholipids.

### The *Phaeobacter* P-stress response reveals a high investment in organic P utilization

To identify the mechanism of degradation, import, and catabolism of the phospholipid headgroups, *Phaeobacter* cultured under P_i_, −P, PE, and PC growth conditions was subjected to comparative cellular proteomics. In cellular fractions, P limitation induced a large proteomic response that was attenuated when cells were supplemented with the lipid headgroups consistent with their use as a sole P source (Table 1 and table S2). The P stress response included increased synthesis of the two-component regulatory system (PhoBR), the high affinity P_i_ ABC transporter (PstSABC), C-P lyase components, various extracellular phosphomonoesterases and phosphodiesterases, and putative organic P transporters (Table 1). The intracellular phospholipase C (PlcP), required for lipid remodelling, was also differentially synthesized in P-deplete *Phaeobacter* cultures. Construction of a Δ*phoB:Gm* mutant confirmed that most of these proteins belonged to the *Phaeobacter* Pho regulon (Table 1). Subsequent comparative genomics analysis showed the *Phaeobacter* Pho regulon to be more extensive compared to other marine *Alphaproteobacteria*, suggesting that this bacterium is specialized at scavenging organic P moieties (table S3).

**Table 1.** The P-stress response of *Phaeobacter* sp. MED193 wild type and *ΔphoB* cultures, highlighting proteins of the Pho regulon. A positive fold change (FC) indicates a higher detection compared to the P_i_ control, whereas negative folds indicate a lower detection, and N/D marks nondetectable proteins. The relative abundance (%) highlights substrate-binding proteins (PstS and PhnD) and phosphatases (PhoX and GlpQ1) that are essential for the P-stress response. ATPase, adenosine triphosphatase; ABC, ATP-binding cassette.

				Wild type			*∆phoB*		
Locus tag	UniProt_ID	Annotation			−P	%		−P	%
MED193_03127	A3XC77	UshA	Phosphoesterase, 5′-nucleotidase	++*	4.03	0.75		−0.65	0.01
MED193_04037	A3XBP5	PhoB	Transcriptional regulatory protein	++	4.30	0.05		N/D	N/D
MED193_04042	A3XBP3	PhoU	Phosphate uptake regulator	++	3.09	0.37		−0.17	0.00
MED193_04047	A3XBP1	PstB	ATPase component of ABC transporter	++	3.40	0.30		−0.45	0.00
MED193_04052	A3XBP2	PstA	Permease component of ABC transporter	++	4.32	0.12		N/D	N/D
MED193_04057	A3XBN9	PstC	Permease component of ABC transporter	++	7.01	0.06		N/D	N/D
MED193_04062	A3XBP0	PstS	Substrate binding component of ABC transporter	++	4.35	5.96		N/D	N/D
MED193_04067	A3XBN7	PhoR	Sensor histidine kinase	++	4.50	0.04		N/D	N/D
MED193_05784	A3X8H3	PhoX-type I	Extracellular phosphatase	++	4.25	2.24		-0.45	0.00
MED193_06579	A3X815	GlpQ-1	Extracellular glycerophosphoryl diester phosphodiesterase	++	4.55	3.37		N/D	N/D
MED193_07818	A3X9L3		Putative G3P ABC transporter, periplasmic substrate binding protein	++	3.43	0.01		N/D	N/D
MED193_07883	A3X9K3	GlpQ-2	Putative extracellular glycerophosphoryl diester phosphodiesterase	++	2.56	0.01		N/D	N/D
MED193_07888	A3X9J7	UgpC	Putative G3P ABC transporter, ATPase	++	3.55	0.07		−0.72	0.00
MED193_07903	A3X9J4	UgpB	Putative G3P ABC transporter, periplasmic substrate binding protein	++	4.05	0.95		-2.32	0.00
MED193_10151	A3XFY0	PhnE	C-P lyase, permease	++	6.82	0.04		0.21	0.04
MED193_10156	A3XFY1	PhnE	C-P lyase, permease	++	4.70	0.02		0.30	0.02
MED193_10161	A3XFY2	PhnD	C-P lyase, periplasmic substrate binding protein	++	5.68	3.68		N/D	N/D
MED193_10166	A3XFY3	PhnC	C-P lyase, ATPase	++	6.81	0.10		N/D	N/D
MED193_11288	A3XC83		Predicted phosphodiesterase	++	3.55	0.01		N/D	N/D
MED193_11293	A3XC84		Sugar ABC transporter, periplasmic substrate binding protein	++	4.03	0.47	++	3.27	0.01
MED193_11308	A3XC79		Sugar ABC transporter, ATPase	++	5.47	0.02		N/D	N/D
MED193_11419	A3XFL5	PotA	Spermidine/putrescine transporter, ATPase	++	2.64	0.07		−0.29	0.01
MED193_11424	A3XFK5	PotD	Spermidine/putrescine transporter, periplasmic substrate binding protein	++	2.59	0.70		-0.10	0.10
MED193_11927	A3XFR8	PPK2	Polyphosphate kinase 2	++	3.26	0.21	++	−4.38	0.00
MED193_17359	A3X3R3	PlcP	Phosphodiesterase involved in lipid remodeling	++	4.40	0.01		N/D	N/D
MED193_17364	A3X3R1	BtaA	S-adenosylmethionine-diacylglycerol 3-amino-3-carboxypropyltransferase	++	5.67	0.02		N/D	N/D
MED193_17614	A3X3L3	PhnI	C-P lyase, ribosylation, core complex	++	4.68	0.02		N/D	N/D
MED193_17624	A3X3L1	PhnJ	C-P lyase, CP bond cleavage, core complex	++	2.75	0.01		N/D	N/D
MED193_17634	A3X3K9	PhnL	C-P lyase, supports PhnI	++	4.14	0.02		N/D	N/D
MED193_17644	A3X3K7		Putative C-P lyase DUF1045	++	3.98	0.02		N/D	N/D
MED193_17649	A3X3K4	PhnM	C-P lyase, phosphodiesterase, releases pyrophosphate	++	5.07	0.28		N/D	N/D
MED193_18169	A3X3A1		Polymerase/histidinol phosphatase (N-terminal), PHP (C-terminal)	++	4.57	0.03		N/D	N/D
MED193_18254	A3X384	UshA	UDP-sugar diphosphatase, purine conversion	++	7.36	0.05		N/D	N/D
MED193_19934	A3X531		Ferritin-like, iron containing	++	2.26	0.05		0.18	0.03

*P value < 0.01 (++).

### The extracellular phosphatase, PhoX, is active toward PE and PC and contributes toward cleavage in vivo

**Fig. 2.**
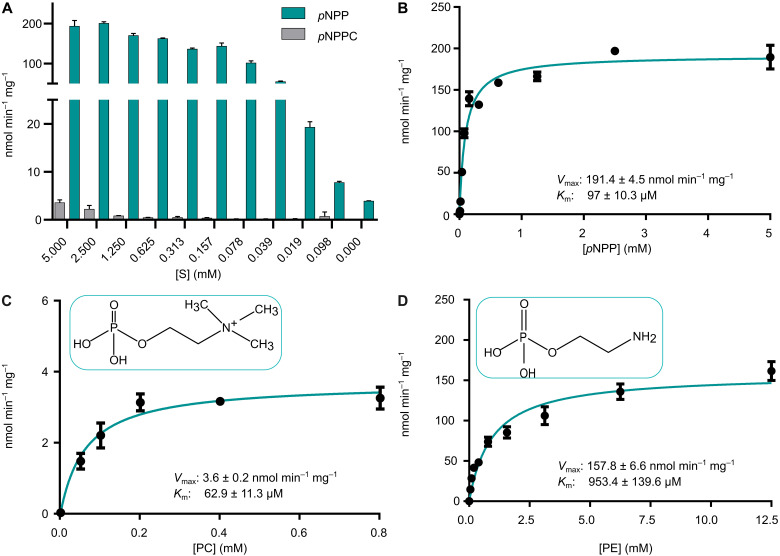
Enzyme kinetics of the PhoX phosphatase on phospholipid headgroups. (**A**) Comparison of the mono- and diesterase activity of PhoX^MED193^ using *p*NPP as the monoester and *p*NPPC as the diester substrate, respectively. Enzyme kinetics of PhoX^MED193^ for (**B**) *p*NPP (*K*_m_: 97 ± 10.27 μM, *V*_max_: 191.4 ± 4.53 nmol min^−1^ mg^−1^), (**C**) PC (*K*_m_: 62.85 ± 11.27 μM, *V*_max_: 3.57 ± 0.16 nmol min^−1^ mg^−1^), and (**D**) PE (*K*_m_: 953.4 ± 139.6 μM, *V*_max_: 157.8 ± 6.60 nmol min^−1^ mg^−1^). Plots show reaction velocity (nmol min^−1^ mg^−1^) against differing substrate concentrations (mM). Lines show fitted Michaelis Menten curves for each substrate. Error bars denote SD of the mean (*n* = 3).

Given that PhoX (MED193_05784) appears to be the sole extracellular phosphatase produced by *Phaeobacter*, and *Phaeobacter* does not have genes to encode the extracellular phosphatases PhoA or PhoD (table S3), we sought to elucidate whether this enzyme could cleave phosphate from the phospholipid headgroups PE and PC. Recombinant His-tagged *Phaeobacter* PhoX (hereafter PhoX^MED193^) was synthesized in a heterologous *Escherichia coli* host and purified using nickel-affinity and size exclusion chromatography. We then used the artificial substrates *para*-nitrophenyl-phosphate (*p*NPP) and *para*-nitrophenyl-phosphorylcholine (*p*NPPC) to determine phosphomonoesterase and phosphodiesterase activity, respectively. PhoX^MED193^ only had phosphomonoesterase activity, while phosphodiesterase activity was barely detectable (Fig. 2A). Like the homologous alkaline phosphatase PhoX from *Pseudomonas fluorescens* (*29*), PhoX^MED193^ requires Fe^3+^ and Ca^2+^ for activity, as shown by electron paramagnetic resonance (EPR) spectroscopy (fig. S2A) and enzyme activity assays (fig. S2B). PhoX^MED193^ liberated phosphate from both PE and PC with varying affinities for each (*p*NPP: *K*_m_ 97 μM; PC: *K*_m_ 62.85 μM; PE: *K*_m_ 953.4 μM) (Fig. 2, B to D).

**Fig. 3.**
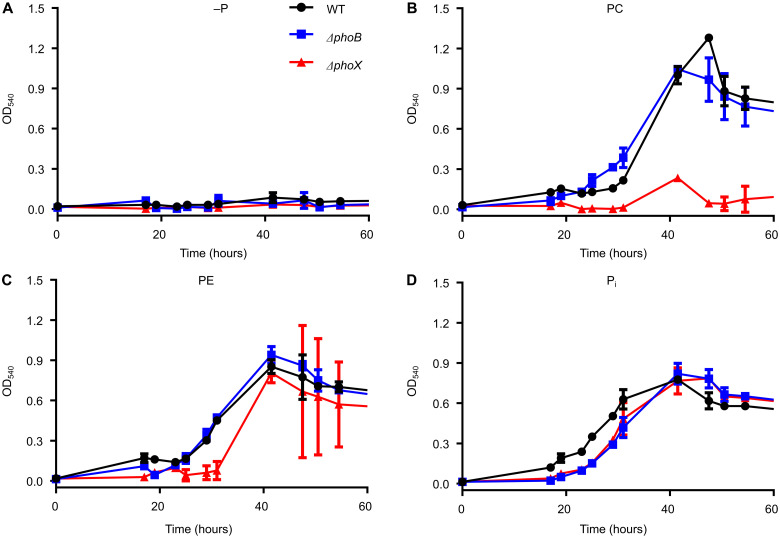
Growth of *Phaeobacter* sp. MED193 wild type, *ΔphoX*, and *ΔphoB* mutants on phospholipid headgroups. Growth (*n* = 3) of prestarved (18 hours) *Phaeobacter* sp. MED193 wild type (WT), *ΔphoB:Gm*, and *ΔphoX:Gm* on low concentrations (50 μM) of (**A**) no P (−P control), (**B**) PC, (**C**) PE, and (**D**) P_i_ as the sole source of P. Error bars denote SD of the mean.

To determine the in vivo role of PhoX in P-lipid utilization, we deleted *phoX* (Δ*phoX:Gm*) and *phoB* (Δ*phoB:Gm*) in *Phaeobacter* and grew both mutants, after 18 hours of P starvation, on varying concentrations (50 μM, 173 μM, and 1.73 mM) of P_i_, PE, or PC as the sole P sources (Fig. 3 and fig. S3). At 173 μM and 1.73 mM concentrations of P, both Δ*phoX:Gm* and Δ*phoB:Gm* still grew on either organic P source, indicating that a *phoB*-*phoX* independent pathway exists (fig. S3). At 50 μM, the *ΔphoX:Gm* mutant had delayed and minimal growth on PC (Fig. 3B). Compared to the wild type, the *phoX* mutant reached similar final cell yields in the cultures grown on PE (Fig. 3C). The *phoB* mutant grew in a similar manner to the wild type, which can be explained by the fact that residual levels of PhoX were detected in the proteomes of this mutant (Table 1).

### Phospholipid degradation in *Phaeobacter* connects the P, N, and C cycles via osmolyte catabolism

**Fig. 4.**
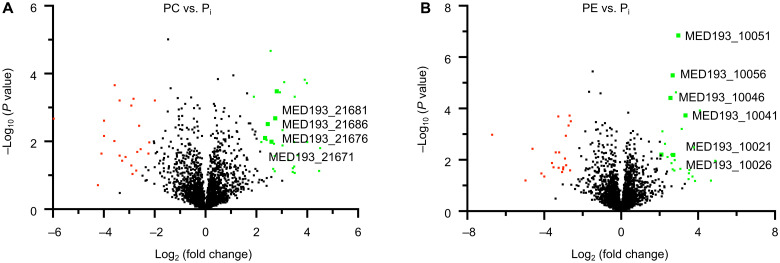
Proteomic response of prestarved *Phaeobacter* sp. MED193 cultures to addition of PC and PE. Volcano plots show the comparison between cultures supplemented with (**A**) PC or (**B**) PE and the P_i_ control. Scatter points represent proteins. The *x* axis is the fold change for the ratio between growth conditions, and the *y* axis is the statistical *P* value. Green dots represent proteins that are significantly up-regulated in PC or PE growth conditions, whereas red dots represent those proteins that are significantly down-regulated.

**Fig. 5.**
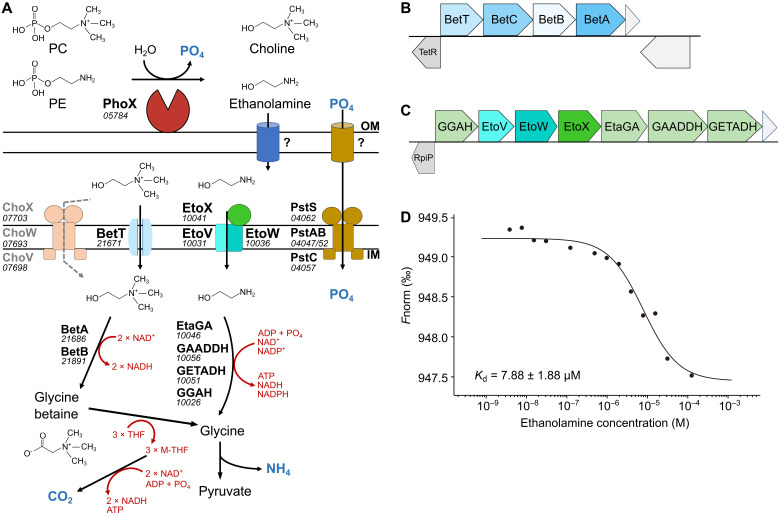
Predicted choline and ethanolamine catabolic pathways in *Phaeobacter* sp. MED193. (**A**) Schematic representation of the degradation pathways. (**B** and **C**) The genomic environment of genes involved in catabolism of (B) choline and (C) ethanolamine in *Phaeobacter* sp. MED193. TetR: transcriptional regulator (MED193_21666); BetT: choline transporter (MED193_21671), BetC: choline sulfatase (MED193_21676); BetB: betaine aldehyde dehydrogenase (MED193_21681); BetA: choline dehydrogenase (MED193_21686); ChoW: ABC-type transporter, permease component (MED193_07693); ChoV: ABC-type transporter, ATP-binding component (MED193_07698); ChoX: ABC-type transporter, betaine/carnitine/choline binding protein (MED193_07703); RpiR: RpiR regulator (MED193_10021); GGAH: γ-glutamylglycine amidohydrolase (MED193_10026), EtoV: TRAP transporter, small permease component (MED193_10031); EtoW: TRAP transporter, large permease component (MED193_10036); EtoX: TRAP transporter, ethanolamine binding protein (MED193_10041); ETAGA: ethanolamine γ-glutamylase (MED193_10046); GAADDH: Γ-glutamylacetyl-aldehydeamide dehydrogenase (MED193_10051); GETADH: γ-glutamylethanolamide dehydrogenase (MED193_10056); PstA: ABC transporter, permease component (MED193_04047); PstB: ABC-transporter, permease component (MED193_04052); PstC: ABC-transporter, ATP-binding component (MED193_04057); PstS: ABC-transporter, P_i_-binding protein (MED193_04062). (**D**) MicroScale Thermophoresis analysis defining binding affinity of MED193_10041 to ethanolamine. Purified protein was mixed with serially diluted concentrations of ethanolamine and binding affinity measured. The *x* axis represents the logarithmic concentration of serially diluted ethanolamine (M); the *y* axis represents the normalized fluorescence (*F*norm). Binding affinity was calculated with *K*_d_ of 7.88 ± 1.88 μM. *n* = 3.

For *Phaeobacter* cells grown on PE and PC as the sole P source, we identified 26 and 30 proteins, respectively, differentially (positively) synthesized compared to the P_i_ control (Fig. 4, Table 2, and table S4). These included proteins required for ethanolamine (PE-grown) and choline (PC-grown) transport and catabolism (Fig. 5A), which were also induced in the *phoB* mutant. Regulation of these proteins is known to be substrate inducible (*30*), demonstrating that hydrolysis of the P-lipid headgroup does occur, liberating the nitrogen-containing alcohol. For PC-grown cells, these included the choline transporter (BetT), betaine aldehyde dehydrogenase (BetB), and choline dehydrogenase (BetA) (Fig. 5, A and B), required to produce glycine betaine (GBT) in *Ruegeria pomeroyi* (*31*), as well as several putative proteins required for converting GBT to glycine (table S4).

**Table 2.** Proteins that are up-regulated in the PC and PE conditions and are involved in the catabolism of both substrates, compared to the P_i_ control. A positive fold change (FC) indicates a higher detection compared to the P_i_ control, whereas negative folds indicate a lower detection. The relative abundance of proteins is given in percent (%).

			−P			PC			PE		
Locus tag	UniProt_ID	Annotation		FC	%		FC	%		FC	%
MED193_10026	A3XG12	γ-glutamylglycine amidohydrolase		−0.26	0.00		−0.42	0.00	+*	2.71	0.01
MED193_10041	A3XG05	TRAP transporter, periplasmic ethanolamine binding protein	+	2.14	0.05		0.10	0.01	++	3.35	0.12
MED193_10046	A3XG06	Ethanolamine γ-glutamylase		1.29	0.02		0.11	0.01	+	2.56	0.06
MED193_10051	A3XG07	γ-glutamylacetylaldehydeamide dehydrogenase		1.20	0.05		0.19	0.02	++	2.97	0.17
MED193_10056	A3XFZ8	γ-glutamylethanolamide dehydrogenase		0.73	0.04		0.04	0.02	++	2.68	0.15
MED193_11439	A3XFK8	GlpR, transcription regulator, G3P regulon repressor		0.25	0.00		−0.30	0.00	+	2.79	0.01
MED193_19144	A3X5J0	Dimethylglycine dehydrogenase (DMGDH)	+	1.77	0.02	+	2.80	0.04		0.01	0.01
MED193_21671	A3XF94	BetT, predicted membrane choline transporter		0.39	0.00	+	2.60	0.00		−1.19	0.00
MED193_21676	A3XF95	BetC, choline sulfatase		1.16	0.00	+	2.34	0.00		0.60	0.00
MED193_21681	A3XF87	BetB, betaine aldehyde dehydrogenase		1.65	0.01	+	2.74	0.01		−0.21	0.00
MED193_21686	A3XF88	BetA, choline dehydrogenase		0.44	0.00	+	2.45	0.00		−0.36	0.00

*P value < 0.05 (+) and <0.01 (++).

For PE-grown cells, the differentially synthesized proteins (Table 2) were encoded by genes from a single operon (Fig. 5C). These genes are closely related to those demonstrated to function as an ethanolamine utilization pathway in *Chromohalobacter salexigens* and *Agrobacterium tumefaciens* c58 (*32*). To determine whether the periplasmic substrate-binding domain (EtoX) of the TRAP-type transporter system (encoded by MED193_10041) also binds ethanolamine like its homolog in *C. salexigens* (*32*), we purified recombinant EtoX protein and undertook affinity assays using microscale thermophoresis and isothermal titration calorimetry (ITC). Recombinant EtoX had a binding affinity (*K*_d_) for ethanolamine of 7.9 ± 1.9 μM, while other substrate analogues tested, e.g., PE, glycerol-1-phosphate (G1P), glycerol-3-phosphate (G3P), and PC, showed no binding (Fig. 5D and table S5).

### Ethanolamine transporter genes are abundant and highly expressed throughout the global ocean

**Fig. 6.**
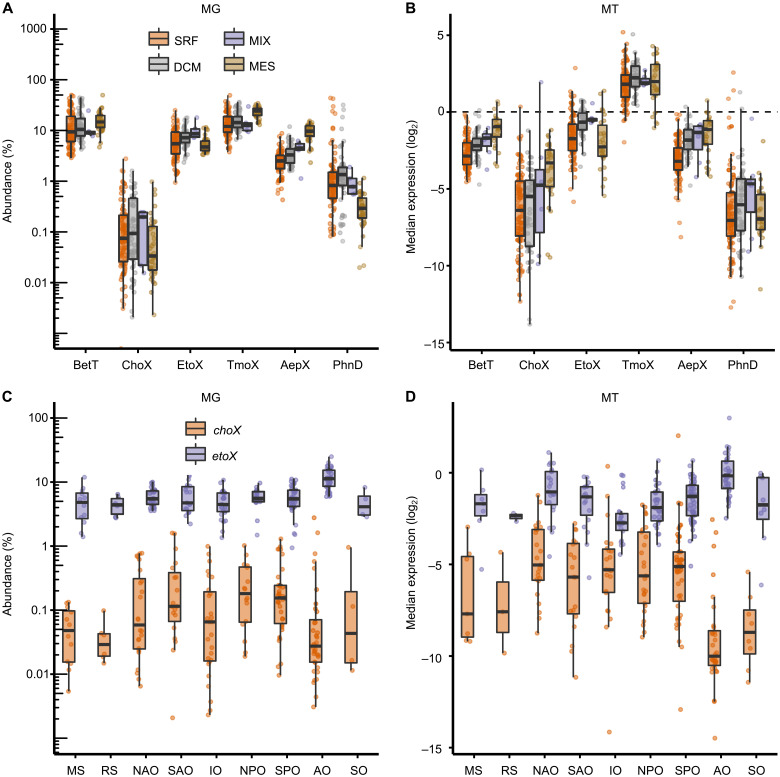
The abundance and distribution of substrate binding proteins in both metagenomes and metatranscriptomes of the Tara Oceans database. Plots are shown as a function of sample depth (**A** and **B**) or oceanic sampling site (**C** and **D**). BetT and ChoX, choline; EtoX, ethanolamine; TmoX, trimethylamine *N*-oxide (TMAO); AepX, aminoethylphosphonate; PhnD, phosphonate. Metagenome (MG) abundance was calculated as a percentage of the median abundance of 10 prokaryotic single-copy marker genes (*40*), whereas metatranscriptome (MT) abundance was calculated as log_2_ transforms of transcript abundance normalized to the median abundance of the same 10 prokaryotic single-copy marker transcripts. DCM, deep chlorophyll maximum; MES, mesopelagic zone; MIX, mixed layer; SRF, surface water; MS, Mediterranean Sea; RS, Red Sea; NAO, North Atlantic Ocean; SAO, South Atlantic Ocean; IO, Indian Ocean; NPO, North Pacific Ocean; SPO, South Pacific Ocean; AO, Arctic Ocean; SO, Southern Ocean.

Given that lipid headgroups present labile C, N, and P sources, we sought to determine the distribution and expression of *etoX* and *betT*, as well as the gene (*choX*) encoding the choline substrate binding protein ChoX (*31*, *33*) in the global ocean using the TARA Ocean metagenomes and transcriptomes (*34*, *35*). As a comparison, we also scrutinized these datasets for the distribution and expression of the gene encoding trimethylamine *N*-oxide substrate binding protein TmoX, previously shown to be highly expressed in seawater (*36*–*39*), as well as incorporating our recent analysis of the genes encoding the phosphonate binding proteins, *aepX* and *phnD* (*11*). We created profile hidden Markov models for EtoX, BetT, ChoX, and TmoX using functionally characterized representatives and very close homologs, to use as a query to search the metagenomes and metatranscriptomes of the Ocean Gene Atlas (OGA) database (*34*, *35*). Retrieved hits were manually checked via sequence alignment and phylogenetic reconstruction, and values were normalized by taking the median abundance of 10 prokaryotic single-copy marker genes/transcripts (*34*, *40*) for four different sampling depths and nine different oceanic sampling sites (Fig. 6). Both *betT* and *etoX* were present in ~5 to 10% of bacteria, like *tmoX* and *aepX*, while *choX* was less common (Fig. 6A). *betT* and *etoX* were also expressed at comparable levels to *aepX*, suggesting a constant source of both choline and ethanolamine exists in the ocean at all depths (Fig. 6B). Approximately 94% of the hits related to *etoX* belong to the *Alphaproteobacteria* (fig. S4A), mostly in an unassigned group, with 19% belonging to the *Pelagibacterales* (SAR11) and 7% belonging to the *Roseobacter* group (fig. S4B). In contrast, only 50% of *tmoX* ORFs belonged to *Alphaproteobacteria*, with ca. 40% belonging to *Gammaproteobacteria* (fig. S5B). Thus, while *etoX* and *tmoX* are both abundant across the ocean, their phylogenetic origin varies, suggesting resource partitioning in metabolic traits.

## DISCUSSION

The exchange of DOM between phototrophs and heterotrophs underpins the marine microbial loop and the functioning of respective food webs (*5*, *41*). Marine DOM comprises complex biopolymers that require specific molecular mechanisms to perform extracellular hydrolysis and subsequent high affinity transport (*38*, *39*, *42*–*44*). Metabolites are then shared in mutualistic or competing relationships (*45*, *46*). Despite the myriad of metabolites comprising the DOM pool (*47*) and the importance of metabolic “sharing” between marine microbes, the molecular mechanisms underpinning the breakdown of these metabolites remain poorly characterized although their mineralization is key to these interactions. To this end, our understanding of phospholipid degradation is very limited, although such molecules are present in all marine organisms and constantly being liberated via cell lysis, grazing, or active release of membrane vesicles (*48*, *49*).

In identifying the degradation pathways for the phospholipid headgroups PC and PE in a marine *Roseobacter*, we show that hydrolysis of the phosphomonoester bond not only produces bioavailable P_i_ but also stimulates mineralization of the organic nitrogen moieties choline and ethanolamine from PC and PE, respectively. Both SAR11 and *Roseobacter* target choline, GBT, and other methylated amines primarily as energy sources, which can result in the subsequent remineralization of ammonium (*30*, *50*). Therefore, we present a cascade of nutrient recycling (Fig. 5A) and the liberation of inorganic nutrients that are required to sustain biological production mediated by phospholipid breakdown.

Phosphatidylethanolamine is a major membrane lipid abundant in all bacteria including cosmopolitan pelagic marine bacteria such as *Pelagibacter ubique* (*14*, *51*, *52*). Phosphatidylcholine is generally found in greater abundance in eukaryotic cells but also found in some bacteria (*53*). However, data on the standing stock concentrations of these lipids across the global ocean are limited (*28*). Furthermore, these measurements do not accurately portray in situ phospholipid turnover rates. If we assume that *etoX* expression is a proxy for phosphatase-dependent PE hydrolysis, then the ubiquitous expression of *etoX* across all oceanic sites and depths, from mesopelagic through to surface waters with maximal expression at the deep chlorophyll maximum (Fig. 6B), suggests that PE turnover is constantly occurring throughout the global ocean at a scale comparable to other well-known nutrients (*27*, *39*, *43*, *46*, *54*). While homologs of *betT* are also abundant and highly expressed, with maximal expression in mesopelagic waters, this BCCT-type transporter is usually nonspecific for various osmolytes, and caution regarding the importance of PC and choline in open ocean waters should be taken (*31*, *52*). *choX* is much more selective for choline, and the expression profile of this transporter supports our previous work (*31*), demonstrating that choline metabolism is restricted to bacteria typically associated with eukaryotic phytoplankton blooms in coastal region (*55*). Unlike that observed for the highly expressed *tmoX* and the less abundant *choX* (fig. S5), *etoX* is predominantly associated with *Alphaproteobacteria* with only 1% of retrieved homologs related to *Gammaproteobacteria* (fig. S4A), despite the latter’s cosmopolitan distribution and possession of substrate-binding proteins for other nutrients (*11*). This may provide support to the idea that distinct metabolite pools are catabolized by microbial consortia with specialized metabolic traits (*56*).

Our data also shows that extracellular hydrolysis, in this case mediated by PhoX, releases P_i_ that is subsequently taken up via PstSABC-facilitating membrane phospholipid restoration, a process not previously recognized (*57*). This presents clear evidence for how organophosphorus, which frequently exceeds P_i_ in the ocean (*9*), helps fulfill cellular P quotas in marine microbes. However, the fact that *Phaeobacter phoX* and *phoB* mutants can still grow on relatively high concentrations of PC and PE provides evidence that Pho-independent pathways exist. Whether this involves yet to be identified Pho-independent phosphatases, like the periplasmic PafA (*58*) or transport of the intact lipid headgroup moiety directly into the cell before further catabolism, remains to be determined. Our proteomics data highlight several transporters and putative phosphatase-like hydrolytic enzymes that may be responsible for this process, and further investigation is required to identify these genetically and biochemically. Despite its role in choline transport (*31*), it is unknown whether BetT can also transport PC. Furthermore, BetC, the sulfatase essential for growth of *R. pomeroyi* DSS-3 (*31*) and *Sinorhizobium meliloti* (*59*) on choline-*O*-sulfate has been shown to catabolize PC to some extent (*59*), which may explain eventual growth in the absence of PhoX. That said, given that low P_i_ concentrations persist across ocean systems, in conjunction with relatively high in situ expression levels of the genes encoding the major nonspecific bacterial phosphatases *phoX*, *phoD*, *phoA*, or *pafA* (*58*, *60*), suggests that these enzymes are likely the major route for phospholipid headgroup catabolism and subsequent production of ethanolamine and choline in the ocean. It should be noted that kinetic data for PhoD and PhoA relating to headgroup monoesters are limited, and this warrants further investigation. However, recent data show that PhoA in marine bacteria also efficiently hydrolyzes phosphodiesters and that this activity can often be comparable in the pelagic ocean (*60*) demonstrating in situ activity driving the remineralizing of phosphate and the production of nitrogenous moieties.

In summary, our study highlights how key DOM components can be sequentially broken down via seemingly distinct metabolic pathways, as the product of one pathway becomes a substrate for another. Specifically, we demonstrate that mineralization of an organic P substrate can lead to the potential remineralization of organic amines into inorganic ammonium, strengthening the link between colimiting nutrient cycles in the ocean.

## MATERIALS AND METHODS

### Strains and culture conditions

*Phaeobacter* was grown in marine broth (Difco, Detroit, MI) at 30°C with shaking (150 rpm). A 1% (v/v) inoculum of the initial culture was transferred into modified artificial seawater (ASW) medium (*61*) with reduced P concentration (50 μM final concentration) and growth continued for another 24 hours. Modified ASW medium contained 10 mM sodium succinate as a carbon source, vitamins [1 ml liter^−1^ of marine ammonium mineral salts (MAMS) vitamins (*62*)], and 8.8 mM ammonium chloride. To prepare cultures for P starvation, a 12.5% (v/v) inoculum of this overnight culture was centrifuged at room temperature for 10 min at 3220*g*, washed three times with P-free ASW medium, and inoculated into P-free ASW. Cultures were then starved for 48 hours (Fig. 1A) or 18 hours (Fig. 3 and fig. S3) at 30°C with shaking at 140 rpm to deplete P reserves. For growth on phospholipid headgroups, prestarved *Phaeobacter* cultures were inoculated into modified ASW containing either PC (CAS no. 72556-74-2; Sigma-Aldrich), PE (CAS no. 1071-23-4; Sigma-Aldrich), K_2_HPO_4_ (P_i_), or no P (−P control), to reach a final starting OD_540_ (optical density at 540 nm) of 0.015. Different final concentrations of P supplements were used, varying between 50 μM and 1.73 mM. Each growth experiment was conducted in biological triplicates. Samples for proteomics (40 ml) and lipid (1 ml) analysis were taken during exponential growth (after 17.5 hours of growth). Samples were centrifuged at 4°C for 10 min at 3220*g*, and the supernatant was filtered through 0.22-μm pore size filter units (Minisart, Sartorius) and poured into a new falcon tube. The pellets and the supernatant were snap-frozen in dry ice and kept at −20°C until used.

### Proteomics analysis

Triplicate samples (see above) from each growth condition were digested with trypsin (Roche, Switzerland), extracted as previously described (*63*, *64*), and analyzed by nanoliquid chromatography (LC)–electrospray ionization–tandem mass spectrometry (MS/MS) using an Ultimate 2000 LC system (Dionez-LC Packings) coupled to an Orbitrap Fusion MS (Thermo Fisher Scientific). An LC separation of 60 min for exoproteomics and 120 min for cellular proteomics was performed on a 25-cm column before MS/MS analysis using settings as previously described (*65*). The recorded MS/MS spectra were processed using MaxQuant (v1.5.5.1) (*66*). Protein fold change was based on the label-free quantification method [see (*66*)], using default parameters but selecting the “match-between-runs” function. MS spectra were searched against the *Phaeobacter* protein database (A3X963_9RHOB), and Perseus (v1.6.5.0. MPI of Biochemistry) was used for comparative proteomics analysis (*67*). Protein differences between conditions were identified using a two-sample *t* test. Comparisons were made between each condition and the P_i_ culture. Statistical analysis used false discovery rates of 0.01 and 0.05 and a minimal log_2_ fold change of 2. A protein had to be present in every replicate of at least one condition to be considered valid.

### Lipid extraction and analysis

The external lipid standard *N*-dodecanoyl-heptadecasphing-4-enine-1-phosphoethanolamine (sphingosyl-PE, d17:1/12:0. S-PE, Avanti Polar Lipids, USA) was used to determine the relative abundance of PtdGro and DGTS in samples as described in the study of Cífková *et al.* (*68*). Therefore, a final concentration of 25 μM S-PE was added to culture samples before cell pelleting. Lipids were then extracted using a modified Folch extraction method (*69*, *70*) and analyzed by LC-MS as previously described (*69*). Initial lipid class identification was based on intact lipid masses. DataAnalysis 4.1 (Bruker Corp., Billerica, MA, USA) software, included in the Compass DataAnalysis 4.1 software package, was used to identify known lipid classes in the MS spectra (*69*, *71*–*73*). Thereafter, QuantAnalysis (Bruker Corp.) was used to integrate signals of PtdGro, DGTS, and S-PE. The accuracy of the mass/charge ratio value was set to ±0.05, the window for retention time was set to 1 min, and the signal/noise ratio was set at 10 for positive mode (DGTS) and 5 for negative mode (PtdGro). Each area was checked manually and adjusted if required. Integrated signals of PG and DGTS were extracted and normalized by the integrated signal of S-PE. Statistical analyses were performed with one-way analysis of variance (ANOVA) by using three replicates. *P* values: not significant > 0.01, ***P < 0.001, ****P < 0.0001.

### Protein overexpression and purification

The full-length codon optimized MED193_10041 gene encoding EtoX from *Phaeobacter* sp. MED193 was synthesized by the Beijing Genomics Institute (China) and then subcloned into the pET-22b vector with a C-terminal His-tag (Novagen, America). The EtoX protein was expressed in *E. coli* BL21 (DE3). Cells were grown at 37°C in lysogeny broth medium containing ampicillin (100 μg ml^−1^) and induced by adding 0.5 mM isopropyl-β-d-thiogalactopyranoside at 17°C for 15 hours. The protein was purified by Ni^2+^ nitrilotriacetic acid (NTA) resin (Qiagen, Germany) and a Superdex 200 column (GE Healthcare, USA) with buffer containing 10 mM Tris HCl (pH 8.0) and 100 mM NaCl.

The pET151/D-TOPO plasmid containing the full-length gene of PhoX^MED193^ (MED193_05784) with N-terminal His-tag was codon optimized and synthesized via GeneArt (Thermo Fisher Scientific, USA). The plasmid was transformed into *E. coli* BL21 (DE3), and the protein was expressed using a rich self-inducing medium (*74*) and the following protocol: 7 hours at 25°C followed by 18 hours at 18°C. Cell pellets were lysed in lysis buffer [50 mM Hepes, 250 mM NaCl, 5% glycerol, Roche cOmplete ULTRA EDTA-free proteinase inhibitor tablets, Benzonase nuclease, and 1× Merck Millipore BugBuster (pH 8.0)] by sonication and the supernatant applied onto a Roche cOmplete His-Tag purification gravity column, washed with washing buffer [50 mM Hepes, 250 mM NaCl, 5% glycerol, and 10 mM imidazole (pH 8.0)], and eluted with buffer containing 250 mM imidazole. Hereafter, PhoX^MED193^ was further purified by size exclusion chromatography on a HiLoad 16/600 Superdex 200 pg (GE Healthcare, USA) and transferred into activity buffer [50 mM Hepes, 250 mM NaCl, and 5% glycerol (pH 8.0)].

### Microscale thermophoresis

The binding affinity of the purified EtoX protein for ethanolamine was measured using the Monolith NT.115 (NanoTemper Technologies, Germany). The EtoX protein was labeled in assay buffer [1× phosphate-buffered saline with 0.05% (v/v) Tween 20] using the Protein Labelling Kit RED-Tris-NTA (NanoTemper Technologies). For each assay, the labeled protein (about 10 μM) was mixed with the same volume of ethanolamine at 16 different serially diluted concentrations with the highest concentration in the assay being 125 μM. The samples were then loaded into standard capillaries (Monolith NT.115 Capillaries, NanoTemper Technologies) and measured at 25°C using 60% excitation power and medium microscale thermophoresis (MST) power. The data were analyzed using the MO. Affinity Analysis v2.3 software (NanoTemper Technologies).

### ITC measurements

Isothermal titration calorimetry (ITC) measurements were performed with a MicroCal PEAQ-ITC (Malvern, United Kingdom) at 25°C. The sample cell was loaded with 250-μl EtoX sample (100 μM), and the reference cell contained distilled water. The syringe was filled with 75-μl ligand (PE, G1P, G3P, or PC) at a concentration of 1 mM. The EtoX protein and the four ligands were kept in the same buffer containing 10 mM Tris-HCl (pH 8.0) and 100 mM NaCl. Titrations were carried out by adding 0.4 μl of substrate for the first injection and 1.5 μl for the following 14 injections, with a stirring speed of 800 rpm. The data were analyzed with Microcal PEAQ-ITC analysis software.

### Electron paramagnetic resonance

All PhoX^MED193^ EPR samples were prepared in activity buffer (pH 8.0). Two hundred fifty–microliter samples of purified PhoX^MED193^ at 190 μM were prepared with additives at final concentrations of either 5 mM dithionite, 20 mM dithionite, 50 mM EDTA, 1 mM PE, or no additives (no additive control). PhoX^MED193^ activity buffer was used as a blank. All PhoX^MED193^ samples were measured on a Bruker EMXplus EPR spectrometer equipped with a Bruker ER 4112SHQ X-band resonator at 10 K. Sample cooling was achieved using a Bruker “Stinger” cryogen free system mated to an Oxford Instruments ESR900 cryostat, and temperature control was maintained using an Oxford Instruments MercuryITC, as reported previously (*75*–*77*). The EPR spectra were measured with a microwave power of 20 dB (2.2 mW), a modulation amplitude of 5 G, a time constant of 82 ms, a conversion time of 12 ms, a sweep time of 120 s, a receiver gain of 30 dB, and an average microwave frequency of 9.385 GHz, as previously described (*29*). Each spectrum was averaged over four to six scans to get a better signal to noise ratio. The analysis of the continuous wave EPR spectra was performed using EasySpin toolbox (5.2.28) for the MATLAB program package (*78*).

### Metal requirements of PhoX^MED193^

To determine the metal requirements of PhoX^MED193^, 25 ng of protein per 100 μl was supplemented with 1 mM metal solution [H_2_BO_3_, MnCl_2_, ZnSO_4_, Na_2_MoO_4_, CuSO_4_, and Co(NO_3_)_2_] and 1 mM *p*NPP, loaded into a 96-well microplate, and incubated at 30°C, and color changes were noted (see *p*NPP assay details below). Each metal was tested either alone or in combination with a second metal.

### Phosphatase assays using chromogenic and nonchromogenic substrates

The *p*NPP assay was carried out in activity buffer. However, the PiPer assay (Invitrogen), required for organic P-compounds, required a specific reaction buffer [100 mM Tris-HCl (pH 7.5)]. For each reaction, a final concentration of 100 mM CaCl_2_ and 25 ng of PhoX^MED193^ was used. Serially diluted *p*NPP (9.8 μM to 5 mM) and CaCl_2_ were added to PhoX^MED193^, the plate was incubated for 1 hour at 30°C in a microplate reader, and color change was measured by continuously measuring OD_405_. Samples without enzyme, with denatured enzyme, and without substrate were used as negative controls. For the PiPer assay (Invitrogen), a modified working solution [reaction buffer, 100 μM Amplex red solution, Maltose phosphorylase (4 U ml^−1^), glucose oxidase (2 U ml^−1^), horseradish peroxidase (0.4 U ml^−1^), and 0.4 mM Maltose], serially diluted PE and PC (98 μM to 62.5 mM) solutions, and 50 ng of PhoX^MED193^ in reaction buffer were loaded into a 96-well microplate in the following chronological order: A: 100 μl of reaction buffer (blank) and B: (i) 50 μl of protein solution or 50 μl of reaction buffer (negative control) + (ii) 10 μl of working solution or 10 μl of reaction buffer (negative control) + (iii) 40 μl of PE/PC or 40 μl of reaction buffer (negative control). The plate was incubated in a microplate reader for 1 hour at 30°C, and color change was monitored by continuously measuring OD_575_.

### Calculation of enzyme kinetics

Readouts from samples were corrected by the blank and negative controls (background fluorescence). Slopes of the reactions were calculated using the simple linear regression function in GraphPad Prism. The specific enzyme activity was calculated considering the reaction time, the standard curve equation, and the protein concentration. Resulting values were plotted against the substrate concentration to create a Michaelis-Menten enzyme kinetics curve in Prism. In addition, Prism was used to calculate *V*_max_ and *K*_m_ of PhoX^MED193^ for *p*NPP, PC, and PE. Triplicates were used for each reaction.

### Construction of *Phaeobacter phoB* and *phoX* deletion mutants

Δ*phoB:Gm* and *ΔphoX:Gm* deletion mutants were generated using a conjugation protocol as previously described (*39*). Briefly, upstream and downstream regions spanning either *phoB* or *phoX* were amplified by PCR (table S1) and cloned into the suicide vector pK18*mobsacB* along with the gentamycin resistance cassette. Plasmids were mobilized into *Phaeobacter* via conjugation with *E. coli* S17 λ*-pir*. Double homologous recombination events were identified by screening transconjugants for their resistance to gentamycin and sensitivity toward kanamycin.

### Biogeography of choline and ethanolamine utilization in the global ocean

The sequences of MED193_10041 (EtoX), Csal_0678 (*32*), and 10 close homologs were used to create a profile HMM. For ChoX, BetT, and TmoX, bona fide sequences previously identified in the study of Lidbury *et al.* (*31*, *39*) were used to generate profile HMMs. These were then used to scrutinize the OGA metagenome and metatranscriptome databases (*34*, *35*). A threshold of *e*^−70^, *e*^−80^, and *e*^−80^ was used for ChoX, BetT, and TmoX, respectively, again guided by our previous work (*31*, *39*). A manually determined *e*-value cutoff of *e*^−60^ was used to ensure homologs clustered with MED193_10041 and Csal_0678 in a phylogenetic tree (376 hits, fig. S1) (*79*–*88*). Here, other members of the TRAP transporter family were added to demonstrate the distinct nature of the ethanolamine-binding protein. In addition, we used multiple sequence alignment to confirm the conserved amino acids in the ethanolamine binding site in all homologs retrieved, as described by Vetting *et al.* (*32*, *81*, *82*). To compare gene/transcript abundance across sites, abundance was normalized using the median gene/transcript abundance of 10 single copy essential genes as per Murphy *et al*. (*11*). Gene abundance was expressed as a percentage, whereas transcript abundance was expressed as log_2_ of normalized values.

## Notes

View/request a protocol for this paper from *Bio-protocol*.
