# Supplementary material for: Bacterial catabolism of membrane phospholipids links marine biogeochemical cycles
Source: Sci Adv. 2023 Apr 26;9(17):eadf5122. doi: 10.1126/sciadv.adf5122 (PMC10132767; doi:10.1126/sciadv.adf5122)
Supplement: Supplementary file 1 — Figs. S1 to S5 Tables S1 and S5 Legends for tables S2 to S4 [file sciadv.adf5122_sm.pdf]

Science Advances
AAAS

Supplementary Materials for

Bacterial catabolism of membrane phospholipids links marine biogeochemical cycles

Linda M. Westermann *et al.*

Corresponding author: David J. Scanlan, d.j.scanlan@warwick.ac.uk

Sci. Adv. **9**, eadf5122 (2023)
DOI: 10.1126/sciadv.adf5122

The PDF file includes:

Figs. S1 to S5
Tables S1 and S5
Legends for tables S2 to S4

Other Supplementary Material for this manuscript includes the following:

Tables S2 to S4

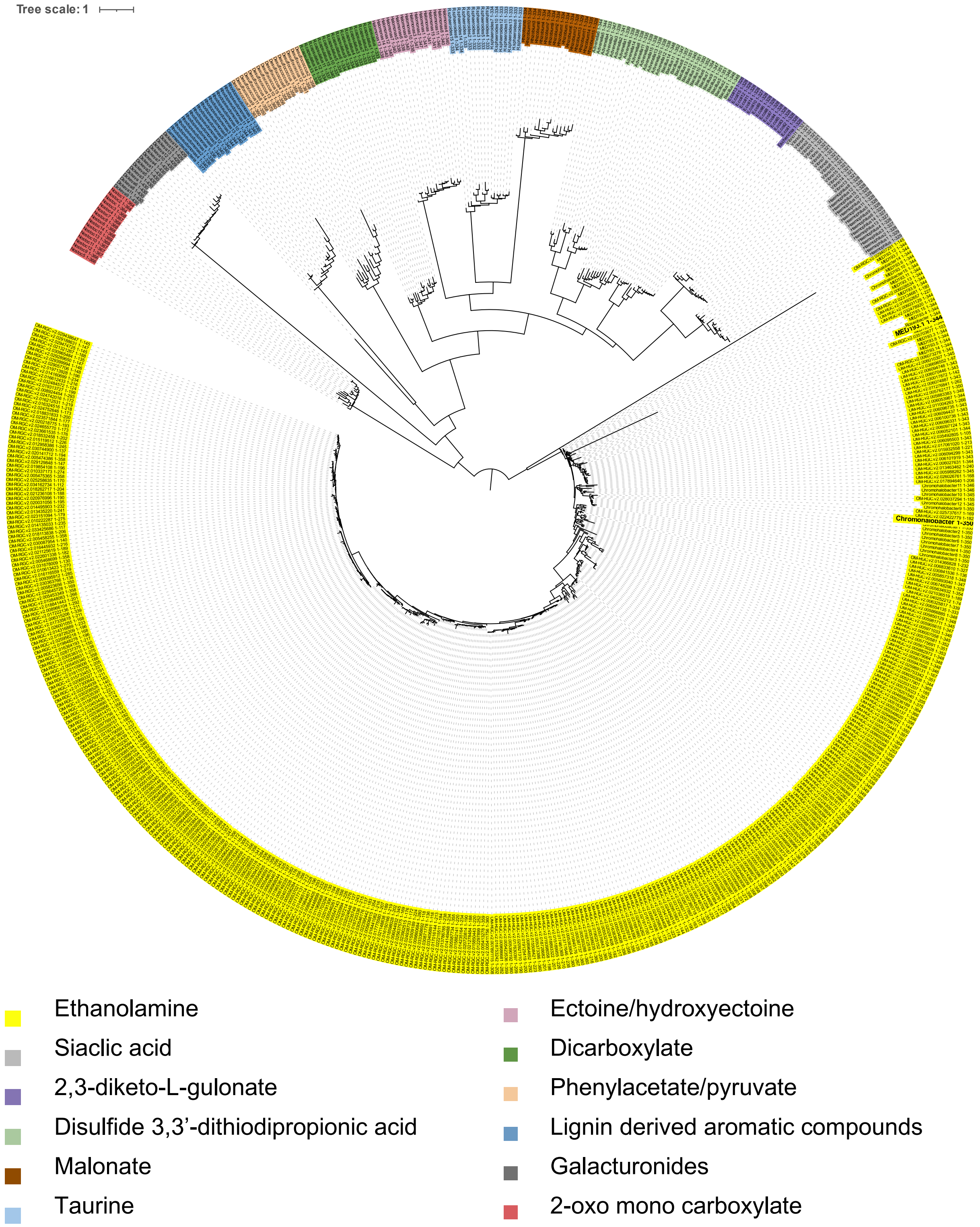

Tree scale: 1
Ethanolamine
Siaclic acid
2,3-diketo-L-gulonate
Disulfide 3,3'-dithiodipropionic acid
Malonate
Taurine
Ectoine/hydroxyectoine
Dicarboxylate
Phenylacetate/pyruvate
Lignin derived aromatic compounds
Galacturonides
2-oxo mono carboxylate

Fig. S1.

Phylogenetic tree of characterised TRAP-T family members and homologs of EtoX (MED193_10041) and Csal_0678. The phylogenetic tree represents characterised members of the TRAP-T family including 10 of their closest homologs obtained through BLAST. Furthermore, the OGA database was searched for homologs of EtoX and Csal_0678 with an e-value cut-off of e^{-60} (*34, 35*). The tree shows a clear clustering of ethanolamine binding proteins found in environmental samples, representing a new member of the TRAP-T family (highlighted in yellow). Tree branches are labelled with the substrate each protein binds to. Branch length represents the expected number of substitutions per site.

A

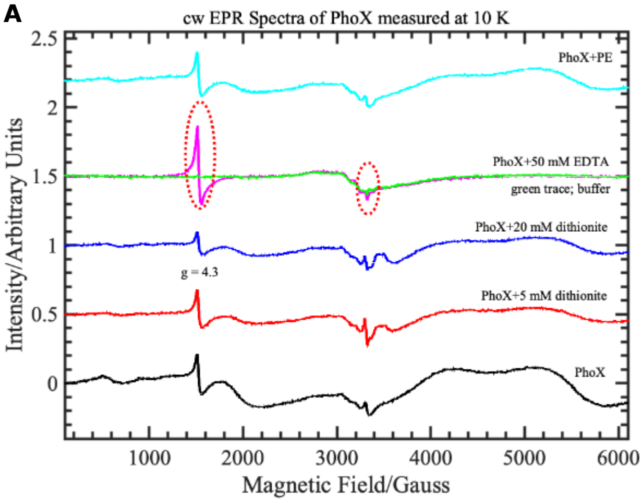

B

PhoX +	$CaCl_2$	buffer
$CaCl_2$	o	o
$ZnSO_4$	x	x
$MnCl_2$	o	x
$NiCl_2$	o	x
$CoCl_2$	o	x
Na_2MoO_4	o	x
H_3BO_3	o	x
Na_2SeO_3	o	x
$CuCl_3$	o	x
Buffer	o	x

Fig. S2.

Metal requirements of PhoX^{MED193}. (**A**) cw-EPR spectra of PhoX^{MED193}. Native protein is expected to have a binuclear Fe^{3+}- Fe^{3+} cluster and no EPR signal (*29*). The spectra of the as-isolated protein (black trace) show the presence of high-spin, ferric ion (EPR signals indicated by the red dotted ellipses), suggests loss of one of the Fe^{3+} ions. EPR signal decreased with increasing dithionite concentration (red and blue traces), reducing Fe^{3+} to the EPR silent Fe^{2+}. The addition of EDTA to the native PhoX^{MED193} enhanced the intensity of the high-spin, ferric EPR signal (green trace), consistent with a previous report (*29*). The addition of phosphorylethanolamine (PE, cyan trace) did not influence the EPR signal, and the substrate does not bind to the Fe^{3+}-centre. The broad EPR signals observed from 3800 G - 5600 G are likely due to the paramagnetic O_2 signal, which is present in all the PhoX^{MED193} variants measured here. **(B)** Colorimetric phosphatase activity assay with *p*NPP. The addition of $CaCl_2$ could restore PhoX^{MED193} activity in the native protein and in combination with all metals tested, except $ZnSO_4$. PhoX^{MED193} + $CaCl_2$: native protein supplemented with $CaCl_2$, PhoX^{MED193} + buffer: native protein. Rows list all metals tested. **o**: release of *p*NP (PhoX^{MED193} activity), **x**: no *p*NP release (no PhoX^{MED193} activity).

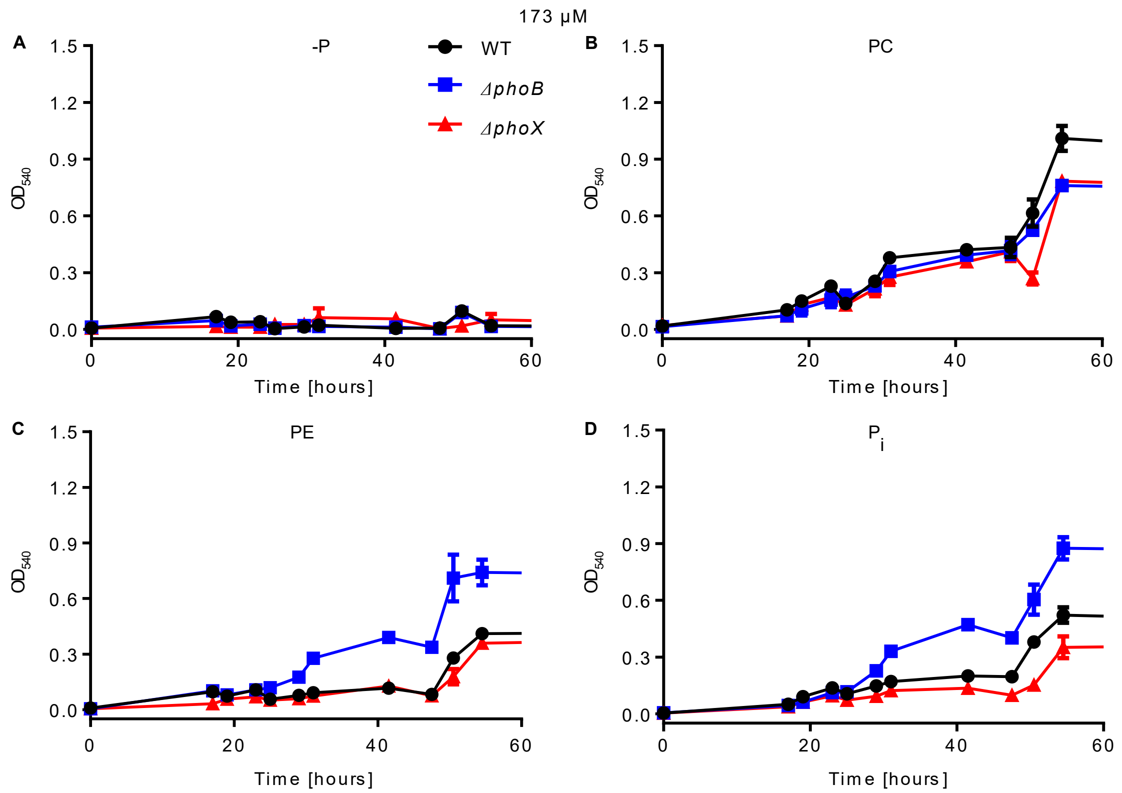
173 µM
A
-P
B
PC
C
PE
D
P_i
WT
ΔphoB
ΔphoX
OD_{540}
0.0
0.3
0.6
0.9
1.2
1.5
Time [hours]
0
20
40
60

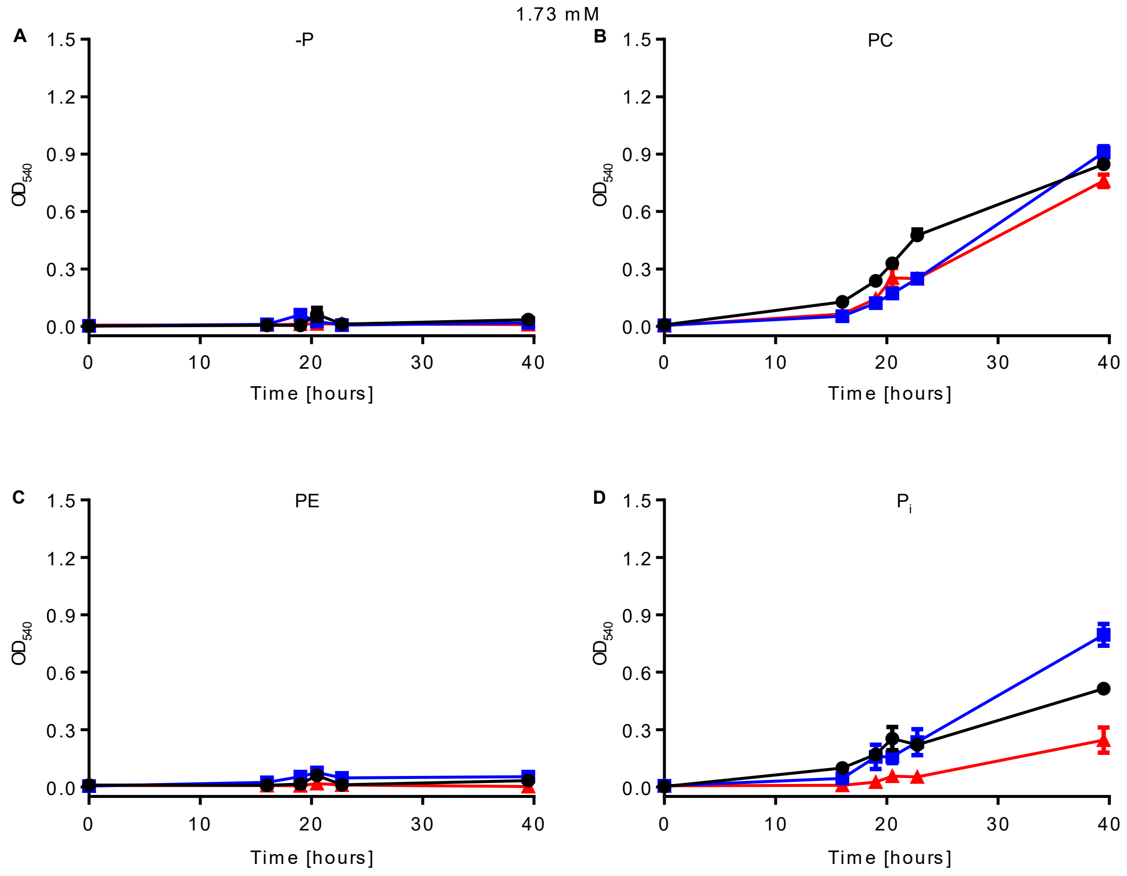
1.73 mM
A
-P
B
PC
C
PE
D
P_i
OD_{540}
0.0
0.3
0.6
0.9
1.2
1.5
Time [hours]
0
10
20
30
40

Fig. S3.

Growth of *Phaeobacter* sp. MED193 when utilising phospholipid headgroups as the sole P source. Growth (n=3) of pre-starved *Phaeobacter* sp. MED193 wild type, *ΔphoB*, and *ΔphoX* on **(A)** no P (-P control) and 173 µM and 1.73 mM of **(B)** phosphocholine (PC), **(C)** phosphorylethanolamine (PE), or **(D)** P_i as the sole source of P. Error bars denote standard deviation of the mean.

A

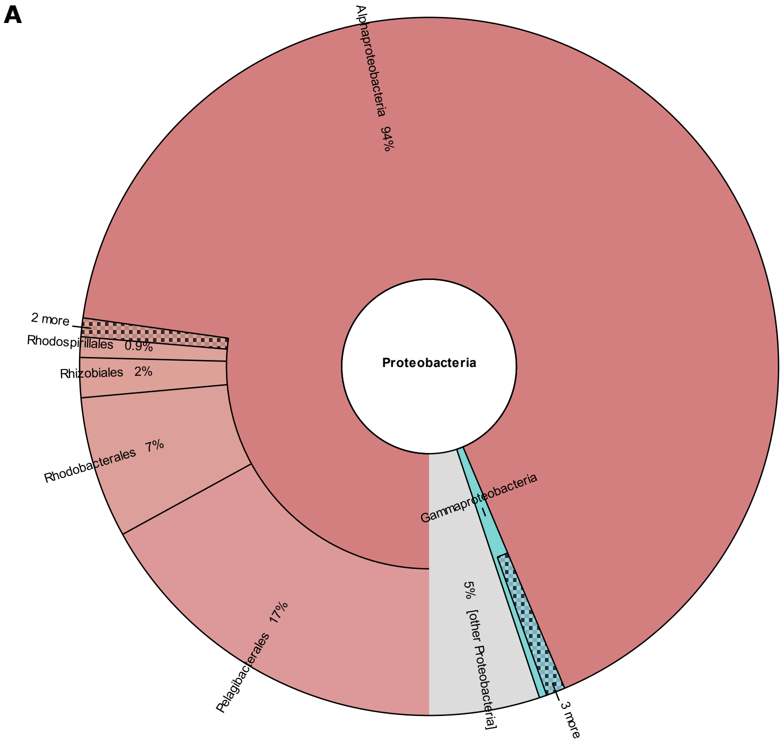

B

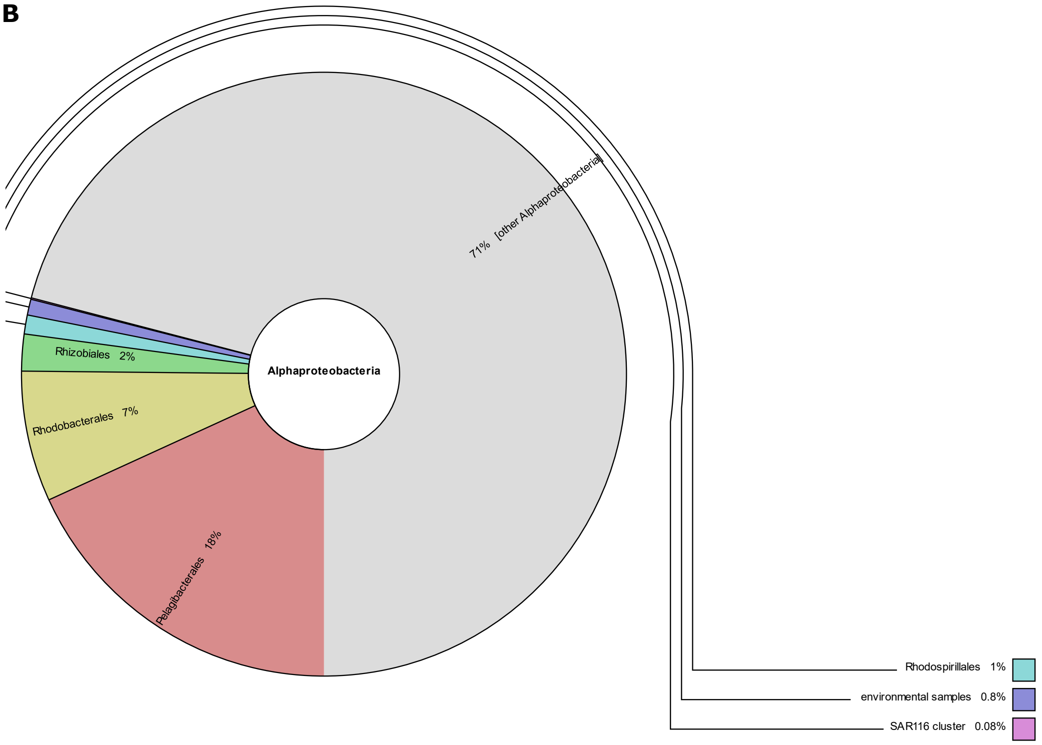

Fig. S4.

Krona plots displaying the distribution of EtoX (MED193_10041) homologs within (A) Proteobacteria and (B) Alphaproteobacteria. Plots were generated using the OGA interphase (*34*, *35*).

A

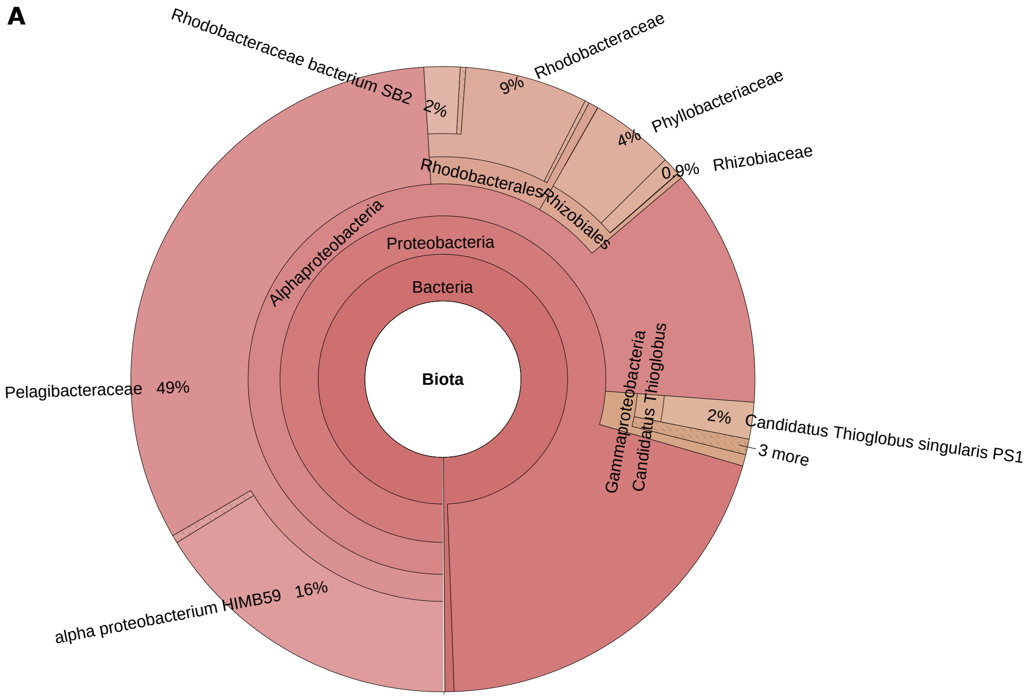

B

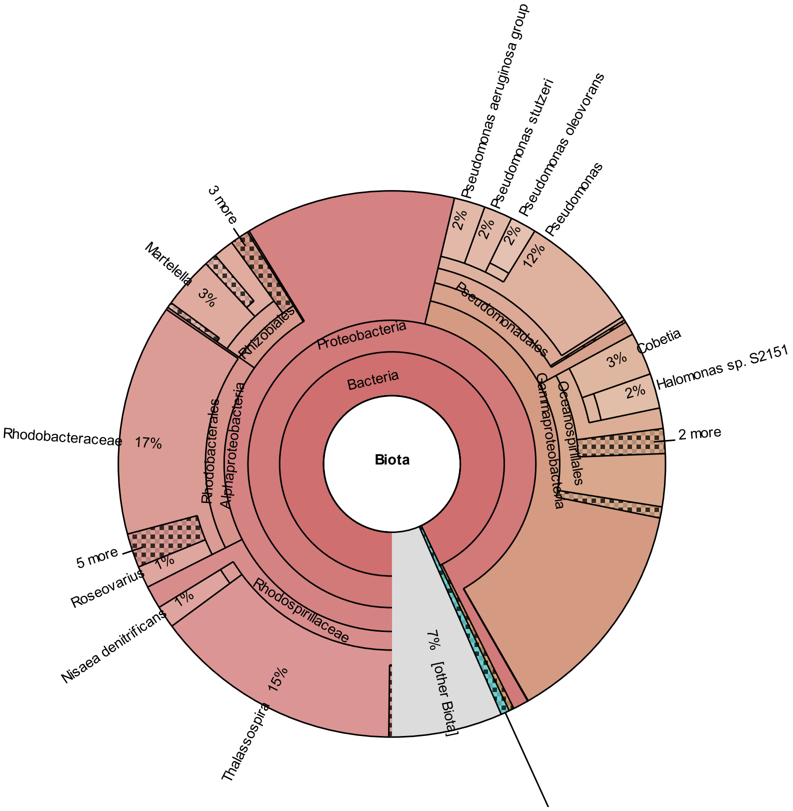

Fig. S5.

Krona plots displaying distribution of (A) ChoX and (B) TmoX homologs within the OGA database.

Plots were generated using the OGA interphase (*34*, *35*).

Table S1.

All PCR primers used for mutagenesis in this study.

Primer	Sequence	Used for
PhoB_AF	tacgaattcgagctcggtacccggggtaacctggagcgtattgg	Cloning region A of MED193_04037 (*phoB*)
PhoB_AR	tctagagtcgacaagccttcttcgccgttttc	
PhoB_BF	acgccgcggccaacagtttggtggtgctgatc	Cloning region B of MED193_04037 (*phoB*)
PhoB_BR	cgttgtaaaacgacggccagtgccactcttgccggtttgtagc	
PhoB_GF	gcgaagaaggcttgtcgactctagaggatccccgg	Cloning region of Gentamicin cassette
PhoB_GR	accaccaaactgttggccgcggcgttgtga	
PhoB_CONF	aattgtcgattcagcacggg	Confirmation of *ΔphoB:Gm*
PhoB_CONR	tcaatatgaccacgcgcttg	
PhoX_AF	tacgaattcgagctcggtacccgggctcccattccttttctgc	Cloning region A of MED193_05784 (*phoX*)
PhoX_AR	ctctagagtcgacaggtttggattggtcggg	
PhoX_BF	acgccgcggccaacacagatggaactaaggc	Cloning region B of MED193_05784 (*phoX*)
PhoX_BR	cgttgtaaaacgacggccagtgccaagtccctctgccttaactg	
PhoX_GF	caatccaaacctgtcgactctagaggatccccgg	Cloning region of Gentamicin cassette
PhoX_GR	gttccatctgtgttggccgcggcgttgtga	
PhoX_CONF	cgaccacaagagcctgtttt	Confirmation of *ΔphoX:Gm*
PhoX_CONR	acctgtaccgcgtagtcatt	

Table S2 (see attached file).

The P-stress response of *Phaeobacter* sp. MED193 wild type and *ΔphoB* cultures. The Table shows all proteins that were upregulated in either wild type or *phoB* mutant. *p*-values < 0.05 (+), < 0.01 (++).

Table S3 (see attached file).

Comparative genomics of the *Phaeobacter* sp. MED193 Pho regulon with other marine Alphaproteobacteria.

Table S4 (see attached file).

All proteins that are upregulated in the PC and PE growth conditions. p-value < 0.05 (+), < 0.01 (++).

Table S5.

Dissociation constant (K_d) of EtoX towards different substrates.

K_d (μM)	Ethanolamine	Phosphoryl-ethanolamine	Glycerol-1-phosphate	Glycerol-3-phosphate	Phosphocholine
EtoX	7.88 ± 1.88	-	-	-	-

"-", not detectable.
